# Trajectory of health-related quality of life during the last year of life in patients with advanced non-small–cell lung cancer

**DOI:** 10.1007/s00520-022-07359-x

**Published:** 2022-09-16

**Authors:** Are Kristensen, Bjørn Henning Grønberg, Øystein Fløtten, Stein Kaasa, Tora Skeidsvoll Solheim

**Affiliations:** 1 Department of Clinical and Molecular Medicine, Faculty of Medicine and Health Sciences, NTNU-Norwegian University of Science and Technology, N-7491 Trondheim, Norway; 2 Cancer Clinic, St. Olavs Hospital, Trondheim University Hospital, Trondheim, Norway; 3 Department of Thoracic Medicine, Haukeland University Hospital, Bergen, Norway; 4 European Palliative Care Research Centre, Department of Oncology, Oslo University Hospital and Institute of Clinical Medicine, University of Oslo, Oslo, Norway

**Keywords:** NSCLC, HRQOL, Physical functioning

## Abstract

**Background:**

The aims of this study were to assess the trajectory of health-related quality of life (HRQOL) during the last year of life in patients with advanced non-small–cell lung cancer (NSCLC) and to explore when and to what degree deterioration of symptoms and physical functioning accelerate towards the end of life.

**Methods:**

Data from two RCTs of first-line chemotherapy in advanced NSCLC was analyzed. HRQOL was assessed repeatedly using the EORTC QLQ-C30 and LC13. Changes in HRQOL scores were investigated relative to the time of death.

**Results:**

The study sample included 730 patients, with a median of four HRQOL assessments per patient (range 1–9). Fatigue, dyspnea, appetite loss, and cough were the most pronounced symptoms in all phases of the disease trajectory. The deterioration rates of global quality of life, physical function, and key symptoms were relatively slow until 4 months before death. Then, the decline accelerated, and for physical function, fatigue, and dyspnea, there was a very rapid decline in the last 2 months.

**Conclusions:**

Patients with advanced NSCLC experience a high symptom burden that worsens over time, especially in the last 4 months. Regular symptom monitoring may help identify where patients are in the disease trajectory, serve as a trigger for changes in anticancer and symptomatic treatment, and facilitate discussions about end-of-life care.

## Background

Cancer is the second leading cause of death worldwide, accounting for an estimated 10 million deaths in 2020 [1]. For the large number of patients dying of cancer, maintaining quality of life represents a major treatment goal throughout the disease trajectory. Several studies have shown that palliative care concurrent with anticancer treatment contributes to improved symptom management, better quality of life, and less psychological distress at the end of life [2–5]. Hence, international guidelines state that dedicated attention to supportive and palliative needs of patients with advanced cancer should be the standard of care [6, 7].

A key element in integrated models of oncological and palliative care is systematic assessment of patient-reported outcomes (PROs) in terms of symptoms, functioning, and well-being, i.e., essential components of health-related quality of life (HRQOL). PROs are important to identify new or worsening symptoms and should be taken into consideration when choosing and evaluating treatment. Baseline scores and changes in HRQOL are prognostic factors for survival [8–10]. Still, little is known about which changes in HRQOL over time may be expected in patients with advanced cancer, especially towards the end of life.

For health care personnel, increased insight in the course of HRQOL may help assess prognosis, anticipate care needs and identify goals for timely interventions aiming to maintain or improve patients’ quality of life. For patients and their next of kin, information about the disease and its effects is requested in order to deal with their situation [11, 12]. And as the disease progresses, they need to know about which symptoms and functional problems to expect.

The typical “cancer illness trajectory” begins with a period of relatively preserved functional status, followed by a period of marked deterioration and increased symptoms at the end of life [13]. In line with this theory, previous studies in advanced cancer patients have found a marked worsening of functioning and various symptoms in the last months of life [14–18]. However, these studies have predominantly focused on the terminal phase [14, 16], included small and/or heterogeneous patient samples [15, 17, 18], or used assessment tools which evaluate symptoms, but not functioning or overall quality of life [17].

Most cases of lung cancer are diagnosed at an advanced stage, and for patients with metastases, the median survival in population-based studies is less than a year [19, 20]. It has been described that lung cancer patients have more symptoms than other cancer patients [21, 22]. Consequently, a comprehensive analysis of data derived from patients with advanced lung cancer is relevant when trying to understand the pattern and magnitude of changes in symptom burden and functional abilities during the last year of life. The objective of this study was to assess the HRQOL trajectory in the last year of life in patients with advanced non-small–cell lung cancer (NSCLC), using time to death as the point of reference. Furthermore, we examined when and to what degree deterioration of symptoms and physical functioning accelerate towards the end of life.

## Methods

### Patients

We pooled data from two randomized clinical trials (RCTs) comparing first-line chemotherapy regimens in advanced NSCLC. RCT 1 (*n* = 436) compared pemetrexed plus carboplatin (PC) with gemcitabine plus carboplatin (GC) for up to four cycles [23]. RCT 2 (*n* = 437) compared vinorelbine plus gemcitabine (VG) with vinorelbine plus carboplatin (VC) for up to three cycles [24]. Both RCTs were conducted by the same research network, and the eligibility criteria were identical. At inclusion, all patients were chemotherapy naïve and had NSCLC stage IV or IIIB not eligible for curative treatment and WHO performance status (PS) of 0–2. Both trials were approved by ethic committees, and all patients gave written informed consent. In addition to the study treatment, 32% and 43% of patients in RCT 1 and 2, respectively, later received systemic second-line therapy, and 41% and 49% received palliative radiotherapy. Symptomatic treatment and palliative care were provided by local cancer centers according to their local routines.

HRQOL was assessed on the European Organization for Treatment of Cancer (EORTC) Quality of Life Questionnaire (QLQ) Core (C30) and the lung-cancer specific module LC13 at inclusion, after every 3-week cycle of chemotherapy and then every 8 weeks up to week 52 or 57 in RCT 1 and RCT 2, respectively. In both RCTs, survival and HRQOL outcomes between the treatment arms were similar. All patients who were registered as deceased in the RCT database and had completed at least one HRQOL assessment within 365 days prior to death were included in the present study.

### HRQOL measures

The EORTC QLQ-C30 consists of a global quality of life scale, five multi-item function scales, three multi-item symptom scales, and six single-item symptom scales [25]. The LC13 has one multi-item symptom scale evaluating dyspnea and nine single-item scales measuring symptoms commonly associated with lung cancer and its treatment [26]. Scores of both questionnaires were linearly transformed to a scale ranging from 0 to 100 [27]. A high score in global quality of life and on the functioning scales indicates a good health status, while a high symptom scale score represents more symptoms.

### Data analysis

All questionnaires completed during the last year of life were included in the analyses. The assessments were aligned relative to the time of death. For example, month 1 included assessments 1–30 days before death. The mean HRQOL scores within four intervals were then calculated: Less than 3 months before death, 3 to 6 months before death, 6 to 9 months before death, and 9 to 12 months before death. If patients had completed multiple questionnaires within an interval, the average score for that patient was used. The difference in mean HRQOL scores between 9 and 12 months before death and the last 3 months was compared with a mixed linear model with time period as a categorical predictor. The compliance rate was calculated by dividing the number of QLQs completed each month before death with the number of QLQs expected according to the assessment schedules in the RCTs.

We defined a difference in mean scores of 10 points or more as clinically relevant and a difference of more than 20 points as a large difference [28, 29]. The QLQ-C30 scores were compared with age- and gender-adjusted reference values from the general Norwegian population [30, 31]. Since HRQOL was assessed only up to a year after inclusion in the RCTs, sensitivity analyses were performed comparing trajectories for patients with a survival time of less than 12 months and those who lived 12 months or longer.

The change over time in global quality of life, physical function, and the key symptoms fatigue, pain, appetite loss, and dyspnea (LC13) were investigated with mixed linear models, with time before death as the explanatory variable. To test if we could identify time points for accelerated decline, we fitted piecewise models, allowing the change to vary at each month before death. A backward elimination procedure retaining only the significant parameters for the change rate was used to select a more interpretable final model. The level of statistical significance was defined as *p* less than 0.05. All analyses were performed using Stata version 15.1 (College Station, TX, USA).

## Results

### Patient characteristics and HRQOL compliance

Of the 873 patients included in the two RCTs, 767 were deceased at database lock of whom 730 (95%) had completed at least one QLQ in the year before death and was eligible for the present analyses. Median age was 65 years, and 428 (59%) were men (Table 1). Median survival from inclusion in the RCTs was 5.8 months (range 0–25 months). The 730 patients completed a total of 3 183 QLQs, with a median of 4 per patient (range 1–9). The compliance rate decreased gradually from 96% 12 months before death to 80% 3 months before death. In the last 2 months, 75% and 39% of expected QLQs were completed.

**Table 1:** Patient characteristics (*n* = 730)

Characteristic	No	Percent
*Age, years*		
Median (range)	65 (25–90)	
< 65	350	48
65–75	274	38
> 75	106	14
*Gender*		
Female	302	41
Male	428	59
*Survival from inclusion in RCT, months*		
Median (range)	5.8 (0–25)	
< 3	166	23
3–6	211	29
6–9	135	18
9–12	93	12
> 12	125	17
*No. of completed QLQs per patient in the last year of life*		
Median (range)	4 (1–9)	
1	89	12
2	90	12
3	75	10
4	117	16
5	130	18
6	105	14
≥ 7	124	17

### HRQOL trajectories in relation to time to death

The mean global quality of life score was 58 (SD, 20) 9–12 months before death and decreased gradually to 50 (SD, 21) 3–6 months before death (Table 2). In the last 3 months, the mean score was 38 (SD, 21). The mean change from the last 9–12 months until the last 3 months was 20 points (*p* < 0.01). Other scales with a large worsening from the last 9–12 months to the last 3 months were physical, social, and role function (24, 21, and 25 points, respectively) and pain (20 points). Scales with a clinically relevant worsening of 10–19 points were fatigue, appetite loss, dyspnea, constipation, pain in arm/shoulder, or other parts of the body and cognitive function. The mean score trajectories for the 125 patients living longer than 12 months were similar to those for the patients living less than 12 months (*data not shown*).

**Table 2:** Mean HRQOL scores in the year before death (*n* = 730). A high mean score for global quality of life and functioning scales represents good quality of life or high level of functioning, while a high symptom scale score represents more symptoms

	Population reference values	I: 9–12 months to death	II: 6–9 months to death	III: 3–6 months to death	IV: 0–3 months to death	Difference between I and IV^a^
		*n* = 226	*n* = 333	*n* = 507	*n* = 548	
		Mean (SD)	Mean (SD)	Mean (SD)	Mean (SD)	
Global quality of life	73	**58 (20)**	**54 (21)**	**50 (21)**	**38 (21)**	*20*
*Functioning scales*						
Physical	83	**66 (22)**	**63 (24)**	**57 (23)**	**42 (23)**	*24*
Social	82	**66 (26)**	**61 (28)**	**56 (28)**	**45 (30)**	*21*
Role	78	**53 (29)**	**49 (31)**	**43(30)**	**28 (28)**	*25*
Cognitive	83	81 (21)	79 (22)	79 (23)	**71 (26)**	*10*
Emotional	84	74 (21)	74 (22)	**72 (22)**	**66 (24)**	8
*QLQ-C30 symptom scales*						
Fatigue	29	**45 (25)**	**48 (25)**	**53 (25)**	**64 (24)**	*19*
Dyspnea	19	**44 (27)**	**45 (30)**	**48 (30)**	**58 (30)**	*14*
Appetite loss	7	**29 (32)**	**32 (32)**	**37 (33)**	**48 (35)**	*19*
Pain	25	27 (28)	31 (30)	**36 (30)**	**47 (33)**	*20*
Insomnia	23	30 (28)	29 (30)	32 (29)	**36 (30)**	6
Constipation	14	**26 (28)**	**29 (29)**	**33 (31)**	**39 (33)**	*13*
Nausea and vomiting	4	12 (16)	13 (18)	**16 (19)**	**19 (22)**	7
Diarrhea	10	14 (21)	12 (18)	13 (20)	16 (23)	2
Financial difficulties	10	11 (21)	11 (20)	12 (23)	15 (25)	4
*LC-13 symptom scales*						
Dyspnea		38 (23)	39 (25)	41 (25)	51 (26)	*13*
Coughing		35 (25)	37 (26)	38 (26)	41 (27)	6
Pain in arm or shoulder		19 (25)	18 (24)	19 (25)	29 (31)	*10*
Pain in chest		17 (22)	18 (22)	22 (24)	26 (28)	9
Pain in other parts of body		26 (30)	29 (31)	31 (32)	39 (34)	*13*
Peripheral neuropathy		14 (20)	15 (21)	16 (23)	20 (25)	6
Alopecia		15 (26)	15 (26)	15 (25)	17 (26)	2
Dysphagia		9 (18)	11 (21)	12 (22)	16 (24)	7
Sore mouth		8 (15)	11 (20)	10 (18)	14 (21)	6
Hemoptysis		3 (11)	3 (10)	5 (13)	6 (16)	3

Clinically meaningful differences (≥ 10 points) compared to population reference values are marked in bold

^a^A positive value indicates a worsening over time. The difference in mean scores between I (9–12 months to death) and IV (0–3 months to death) were tested using mixed linear models with time period as a categorical predictor. Statistically significant changes considered clinically meaningful (≥ 10 points) are indicated by italic font

Compared to the reference population, the mean scores for global quality of life, physical, social, role and emotional function, fatigue, dyspnea, appetite loss, and constipation were significantly worse (> 10 points) in all time intervals, including 9–12 months before death. For pain and nausea/vomiting, the difference to the reference population became clinically relevant from 6 months before death, and for insomnia and cognitive function in the last 3 months.

### Rates of change in HRQOL towards the end of life

**Fig. 1:**
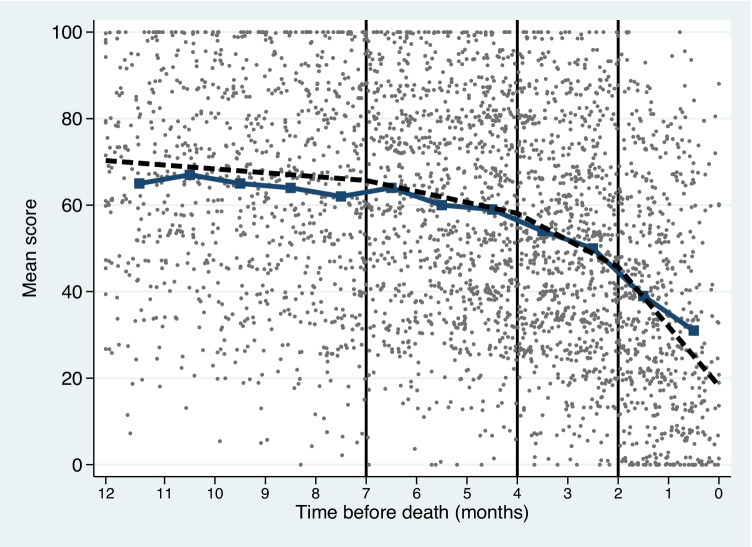
The course of global quality of life during the last year of life. The circles reflect individual data points; the connected line the average scores in each month and the dashed line the estimated values from the piecewise linear mixed model. The deterioration rate increased significantly 4 and 1 month before death

**Fig. 2:**
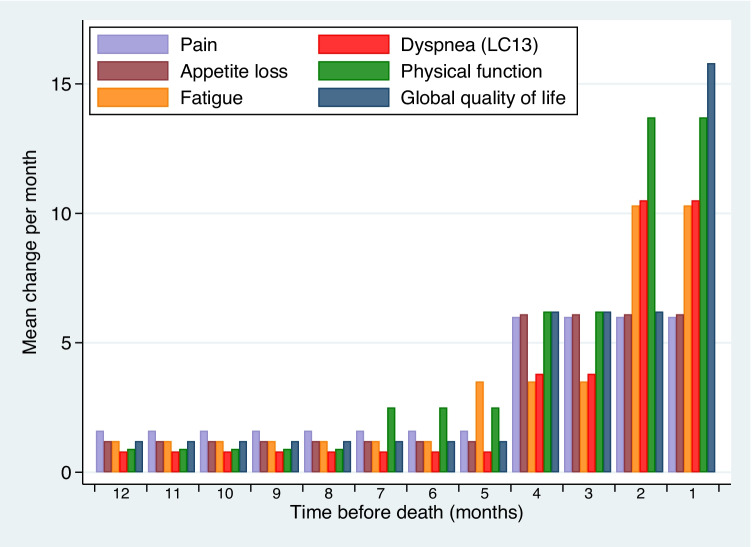
Rate of monthly change in the last year of life for key HRQOL scales, estimated using piecewise linear mixed models. A high value represents a rapid decline

For global quality of life, the mean deterioration rate was 1.2 points/month 12 months before death, with a significant change to 6.2 points/month 4 months before death (Fig. 1). In the last month, the deterioration rate nearly tripled to 15.8 points/month. For physical function and pain, appetite loss, fatigue, and dyspnea, the deterioration was relatively slow (range 1–2 points/month) until 4 months before death (Fig. 2). Later, the decline accelerated, and for physical function, fatigue, and dyspnea (LC13), there was a very rapid decline the last 2 months (range 10–14 points/month).

## Discussion

In this study, patients with advanced NSCLC experienced a substantial deterioration of HRQOL in the last year of life. Fatigue, dyspnea, appetite loss, and cough were the most pronounced symptoms and significantly worse than in the reference population in all phases of the disease trajectory. Notably, mean pain scores were not significantly worse than in the reference population until 6 months before death, but increased thereafter. The ability to carry out physical and social activities was markedly impaired even 9–12 months before death, and then decreased progressively. In contrast, cognitive and emotional functioning was relatively stable during the disease trajectory and only in the last months of life significantly worse than the reference population.

The finding that HRQOL worsens markedly in the last months of life is in line with clinical experience and other studies of cancer trajectories, conducted in more heterogenous patient populations [15–17]. However, comparison of symptomatology across studies is difficult due to differences in the patient samples and assessment strategies employed. In a Swedish study, patients with primary inoperable lung cancer were asked to rank their most distressing symptoms [32]. In all time periods before death, dyspnea, pain, and fatigue were consistently ranked as the most distressing symptoms. Like in our data, these symptoms were also reported as the most prevalent and the mean intensity increased significantly in the last 2 months before death [32].

In clinical practice, symptom deterioration between scheduled hospital visits may go unnoticed. Additionally, clinicians often miss or underestimate symptoms during consultations [33–35], which may further delay timely management. In the present study, the deterioration of key symptoms, physical function and global quality of life was relatively slow until 4 months before death. Then, increased decline was observed, especially in the last 2 months. Possibly, regular PRO monitoring (e.g., weekly or bi-weekly) could identify patients before the worsening has accelerated and the patient’s condition deteriorated. Since salvage therapies are mainly effective in patients with good performance status [36], earlier detection of relapse or disease progression may allow more patients to receive optimal treatment. Indeed, this may be an important mechanism of action in studies of PRO monitoring demonstrating not only improved HRQOL outcomes, but also increased survival [37–40]. Identifying patients with increasing symptoms being ineligible for more anticancer treatment is also important, since these may benefit from dedicated palliative care, including palliative radiotherapy to treat symptoms like pain and dyspnea [41, 42].

The EORTC measures have traditionally been used in research, but can also be used in routine cancer care [43]. Indeed, a recent review found that the QLQ-C30 was the most widely used measure in studies of PRO implementation in clinical practice [44]. A shortened version of the QLQ-C30, the C15-PAL, has been developed for cancer patients with a short life expectancy [45]. In the C15-PAL, the financial difficulties and diarrhea items are excluded, and the nausea/vomiting scale shortened to nausea only. In the current study, these three scales had low average scores during the trajectory, including the last 3 months. In the clinical practice setting, the PRO measures should focus on symptoms that are common, reflect changes in disease status, or are clearly linked with an intervention that could improve them. The results in the present study suggest that for patients with advanced NSCLC, the PAL-15 could be used instead of the QLQ-C30 in clinical practice. These questionnaires, and other PRO instruments, are now available in electronic formats, meaning the patients can complete assessments at home on web-based devices with the results immediately transferred to the medical record [46, 47].

A limitation of the current study is that both RCTs were conducted before the identification of predictive mutations for targeted therapies and the introduction of immunotherapy. However, despite the impressive results reported for these therapies, most patients develop progressive disease, and survival estimates in real-world populations are generally lower than those reported in pivotal clinical trials [19, 20]. Sensitivity analyses indicated that patients whose survival exceeded 12 months had the same HRQOL trajectories in the last period of life as patients with shorter survival. Another limitation is that data on post-study treatment was not recorded in sufficient detail to allow for analyses on how anticancer treatment affected the HRQOL trajectory. Furthermore, inclusion criteria in the RCTs were limited to relatively well-functioning patients (WHO PS 0–2), and the intensity of symptoms and functional problems found in this study may thus represent an underestimation of symptoms experienced in the overall population of patients with advanced NSCLC. Selection of patients with good performance status may also have delayed worsening of symptoms and functioning of patients.

In conclusion, this study shows that patients with advanced NSCLC experience a high symptom burden and significantly impaired quality of life in the last year of life. The degree of worsening increases substantially in the last 2 to 4 months. Regular symptom monitoring may help identify where patients are in the disease trajectory, indicate a need for changes in anticancer and symptomatic treatment, and facilitate discussions about end-of-life care.
